# Inhibition of DOT1L by Half-Selenopsammaplin A Analogs Suppresses Tumor Growth and EMT-Mediated Metastasis in Triple-Negative Breast Cancer

**DOI:** 10.3390/ph14010018

**Published:** 2020-12-28

**Authors:** Woong Sub Byun, Gyu Ho Lee, Hyeung-geun Park, Sang Kook Lee

**Affiliations:** 1 College of Pharmacy, Natural Products Research Institute, Seoul National University, Seoul 08826, Korea; sky_magic@naver.com; 2 College of Pharmacy, Research Institute of Pharmaceutical Sciences, Seoul National University, Seoul 08826, Korea; boomboombibi@snu.ac.kr

**Keywords:** triple-negative breast cancer, metastasis, epithelial-mesenchymal transition, DOT1L, histone methylation, half-selenopsammaplin A analog

## Abstract

Due to a lack of hormone receptors, current treatment strategies for triple-negative breast cancer (TNBC) are limited with frequent disease recurrence and metastasis. Recent findings have suggested that aberrant methylation of histone H3 lysine 79 residue (H3K79me) by the histone methyltransferase disruptor of telomeric silencing 1-like (DOT1L) is a potential therapeutic target for TNBC clinical management. Therefore, we developed DOT1L inhibitors as potential antitumor agents against TNBC cells. We reveal that a synthetic half-selenopsammaplin A analog 9l (subsequently known as 9l) exhibited inhibitory activity against DOT1L-mediated H3K79 methylation, and showed antitumor activity in TNBC cells. The analog 9l also significantly inhibited TNBC invasion and migration via the modulation of epithelial-mesenchymal transition (EMT) markers, including N-cadherin and vimentin downregulation and E-cadherin upregulation. In an MDA-MB-231/Luc-implanted orthotopic mouse metastasis model, treatment with 9l effectively inhibited tumor growth and lung metastasis via DOT1L regulatory activity and EMT processes. Taken together, these findings highlight the potential of 9l as a novel therapeutic candidate for treating metastatic TNBC via DOT1L modulation.

## 1. Introduction

Cancer is a major cause of human death worldwide, and the incidence is gradually increasing triggered by environmental and dietary factors. According to National Comprehensive Cancer Network (NCCN) guidelines, resection surgery is the most common and effective strategy for treating early stages of human cancers, however the recurrence rate can be up to 70% in patients within five years of surgery [1,2]. In women, breast cancer (BC) is not only the most frequently occurring cancer, comprising 30% of overall cases, but it also the second leading cause of cancer-related mortality [3,4]. Most deaths attributed to BC are associated with recurrent and/or metastatic disease. Moreover, recurrent BCs are often highly metastatic, and tend to acquire resistance to previously used conventional therapies, including chemotherapy (i.e., anthracyclines, taxanes, carboplatin, and capecitabine), or targeted bio-drugs (i.e., trastuzumab) [5,6]. BCs are typically categorized into subtypes based on the existence of three surface receptors; i.e., the estrogen receptor (ER), progesterone receptor (PR), and human epidermal growth factor receptor (HER2). Therefore, these receptors are promising therapeutic targets for BC patients. However, approximately 15% of BCs are classified as triple-negative breast cancer (TNBC), where none of these receptors are expressed [7]. Under these conditions, treatment options for TNBC patients are limited. Hormonal therapies and drugs that target HER2 are not effective, and chemotherapy is the only systemic treatment option. Additionally, current chemotherapeutic drugs for BC patients have exhibited a relatively low selectivity for cancer cells, when compared with normal cells [8,9]. Indeed, TNBCs are considered the most aggressive and intractable type of BC, with high recurrence rates and increased metastatic potential. Therefore, novel therapeutic strategies are urgently required to treat patients with metastatic TNBC.

Histone modifications, including methylation, phosphorylation, acetylation, and ubiquitylation, are covalent post-translational modifications (PTM) that play pivotal roles in several biological processes. Recent evidence has demonstrated that the aberrant modulation of histone modification plays a potential role in cancer initiation, progression, and metastasis [10,11,12]. In particular, aberrantly expressed histone methyltransferases (HMTs) in TNBC have been reported to mediate cell development, proliferation, and metastasis and are thus considered novel therapeutic targets for TNBC [13,14]. Among the HMTs, disruptor of telomeric silencing 1-like (DOT1L), a methyltransferase specific for the histone H3 lysine 79 residue (H3K79), was recently reported as mediating the development and maintenance of mixed lineage leukemia (*MLL)*-rearranged leukemia and TNBC metastasis [13,15]. These findings prompted the development of EPZ-5676, a nucleoside-type inhibitor of DOT1L activity, and led to a clinical trial for leukemia. However, in contrast to its efficacy towards leukemia, EPZ-5676 did not show sufficient inhibitory activity against TNBC growth and metastasis [16]. This limited efficacy, therefore, prompted us to explore and develop an effective DOT1L inhibitor against TNBC.

Psammaplin A (PsA) is a disulfide dimer with bromotyrosine groups on both sides of the molecule was first isolated from the marine-derived *Psammaplysilla* sponge in 1987. Since then, PsA has been reported to be an antitumor agent with inhibitory activities towards DNA methyltransferase (DNMT) and histone deacetylase (HDAC). Similarly, in previous studies, we designed and synthesized 116 novel PsA analogs and evaluated DOT1L inhibitory activity in TNBC. Amongst these novel PsA analogs, the heteromonomeric structured analogs were effective in inhibiting DOT1L, while homodimeric structured analogs exhibited effective HDAC inhibitory activities [17,18]. PsA-3091, a heteromonomeric structured analog with a tertiary butyl functional group at the aromatic ring, showed the most effective and selective inhibition towards DOT1L activity, along with suppression of tumor growth and metastasis in TNBC cells [18].

Herein, as part of our ongoing strategy to develop more effective DOT1L inhibitors, we synthesized 14 novel heteromonomeric structural analogs of PsA (half-selenopsammaplin A; HSPA) by replacing the disulfide bond with a selenium-sulfur bond. All analogs exhibited improved antiproliferative activities against TNBC cells, and eight analogs showed significant inhibitory activities against DOT1L. Among the HSPA analogs, a tertiary butyl derivative, 9l, was identified as an effective antitumor candidate for TNBC cells both in vitro and in vivo, displaying potential inhibition towards DOT1L-mediated histone methylation.

## 2. Results

### 2.1. Preparation of Half-Selenopsammaplin A (HSPA) Analogs

**Figure 1:**
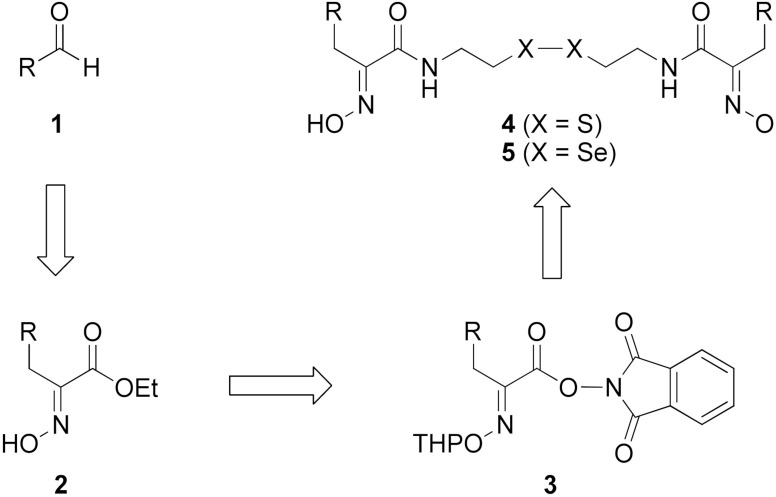
Established synthetic method of psammaplin A analog and selenopsammaplin A analog generation from aldehydes.

**Figure 2:**
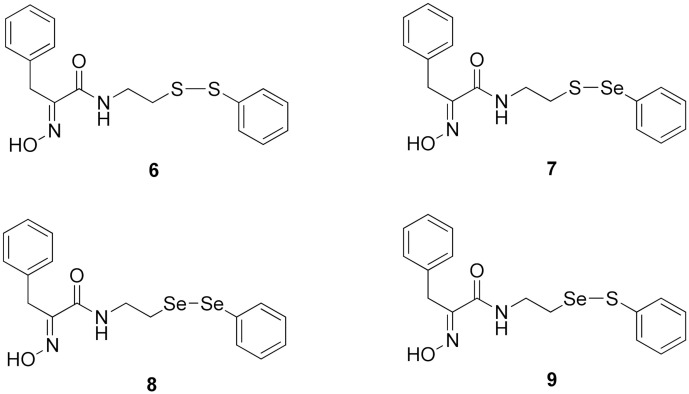
Types of psammaplin A (PsA) heteromonomer derivatives.

**Figure 3:**
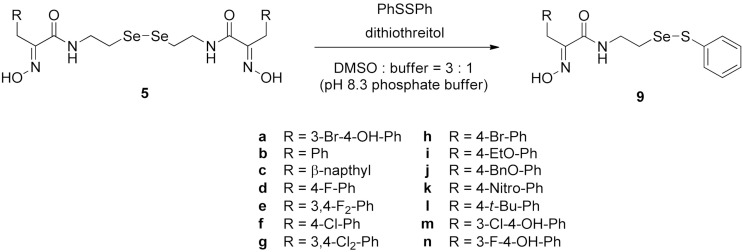
Synthetic half-selenopsammaplin A (HSPA) method and its analogs from various selenopsammaplin A analogs.

Firstly, the synthesis of heteromonomeric psammaplin A analogs was prepared from the analogs of dimeric psammaplin A or selenopsammaplin A (manuscript in preparation) that was previously reported (Figure 1). Before we prepared a series of half monomer analogs, we assessed the molecular stability of four possible analogs (Figure 2). We prepared these analogs using a redox equilibrium process from PsA or HSPA, and diphenyldisulfide or diphenyldiselenide. Amongst these four (**6**–**9**), compound **9** was stable in dichloromethane at room temperature for 48 h (Figure S1). However, the other analogs were slightly decomposed. We finally selected the Se-S analog (half-selenopsammaplin A analog) as a template for monomeric derivatives. Treatment of diphenyldisulfide to a series of selenopsammaplin A analogs in the presence of dithiothreitol (DTT) under dimethylsulfoxide at room temperature successfully resulted the corresponding half-selenopsammaplin A analogs in 40–52% (Figure 3). The chemical structure of half-selenopsammaplin A analogs (**9a**–**9n**) were shown in Table 1.

### 2.2. DOT1L Inhibitory Activity of HSPA Analogs and Expression Level of DOT1L in Human TNBC Cells

**Figure 4:**
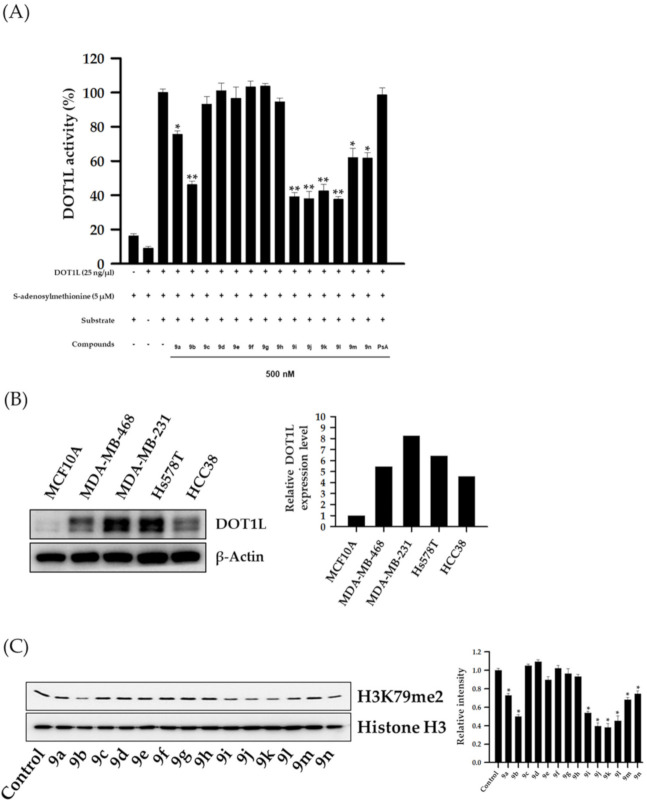
Inhibition of DOT1L enzyme activity by HSPA analogs and DOT1L expression in several TNBC cell lines. (**A**) DOT1L (500 ng/well) enzyme activity was analyzed after incubation with HSPA A analogs (500 nM) for 2 h. All data are expressed as the mean ± standard deviation (SD) (*n* = 3), and are representative of three separate experiments. * *p* < 0.05 and ** *p* < 0.01 indicated statistically significant differences relative to the vehicle-treated control group. (**B**) DOT1L expression levels in several TNBC cell lines were analyzed by western blotting. β-Actin was used as an internal control. The relative intensities of indicated proteins were analyzed semi-quantitatively using National Institute of Health (NIH) Image J 1.52a software. (**C**) MDA-MB-231 cells were treated with 300 nM HSPA analogs for 48 h, and the expression levels of dimethylated-histone H3 lysine 79 residues were determined by western blotting. Histone H3 was used as an internal control. The relative intensities of indicated proteins were analyzed semi-quantitatively using NIH Image J 1.52a software.

To identify small-molecule inhibitors of DOT1L activity, HSPA analogs were evaluated in a cell-free DOT1L enzyme activity assay. Of the tested analogs, eight (**9a**, **9b**, **9i**–**9n**) at a concentration of 500 nM showed significant inhibitory effects against DOT1L enzyme activity, while PsA showed negligible DOT1L inhibition (Figure 4A). Since DOT1L expression levels were highly associated with TNBC progression and metastasis [19], basal DOT1L expression levels were analyzed in several TNBC cell lines (MDA-MB-468, MDA-MB-231, Hs578T, and HCC38). As shown in Figure 4B, all TNBC cell lines exhibited higher DOT1L expression levels when compared to the normal breast epithelial cell line MCF10A. In particular, MDA-MB-231 cells exhibited the highest DOT1L expression levels; therefore, this cell line was used as a TNBC representative in subsequent experiments. Since some analogs exerted inhibitory activities against DOT1L in a cell-free assay, the effects of these HSPA analogs on the expression levels of di-methylated histone H3 lysine 79 residues (H3K79me2, the biological target of DOT1L) were further evaluated by western blotting in MDA-MB-231 TNBC cells. As expected, all eight HSPA compounds effectively suppressed H3K79 methylation in these cells (Figure 4C).

### 2.3. Antiproliferative Activity of HSPA Analogs and Selective Inhibition of H3K79 Methylation in Human TNBC Cells

The antiproliferative potential of HSPA analogs was evaluated in MDA-MB-231 TNBC cells and MCF10A normal breast epithelial cells for 72 h to identify whether HSPA analogs could selectively inhibit the growth of the TNBC cells. As shown in Table 2, all HSPA analogs effectively inhibited MDA-MB-231 proliferation. The antiproliferative activity of novel analogs was up to 17 times higher than that of PsA itself, and up to 12 times higher than previously reported analog PsA-3091. In addition, the growth inhibitory activity of HSPA analogs against MCF10A normal breast epithelial cells was similar to PsA, and significantly lower than paclitaxel. On the other hand, EPZ-5676 was not able to inhibit the proliferation of TNBC cells at the concentration of 50 μM. These findings indicated that the substitution of sulfur to selenium in the disulfide bond of PsA enhanced antiproliferative activities against TNBC cells, without altering effects towards normal cells. Notably, the tertiary butyl derivative, 9l, showed the highest selectivity (therapeutic index value = 161.63) of all analogs. Based on these data, we performed further examination using 9l to elucidate its underlying molecular mechanism toward TNBC cells.

**Figure 5:**
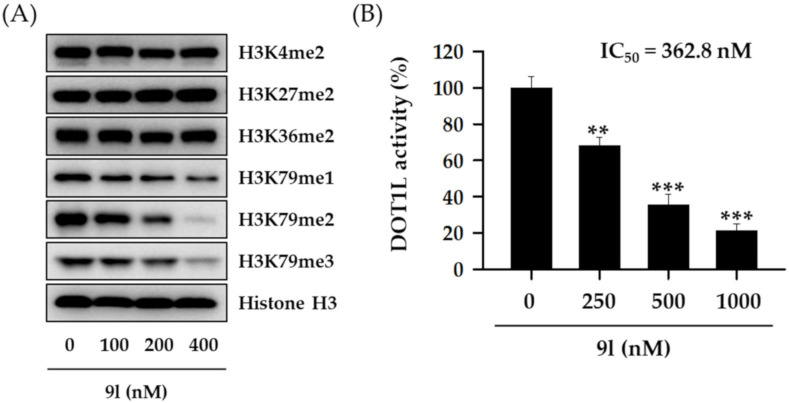
Selective inhibitory activity of 9l towards H3K79 methylation in TNBC cells. (**A**) MDA-MB-231 cells were treated with the indicated concentrations of **9l** for 48 h. The expression levels of various methylated histone H3 lysine residues were then determined via western blotting. Histone H3 was used as an internal control. (**B**) A DOT1L enzyme activity assay was performed using 500 ng/well DOT1L, and incubated with the indicated 9l concentrations for 2 h. All data are expressed as the mean ± standard deviation (SD) (*n* = 3), and are representative of three separate experiments. ** *p* < 0.01 and *** *p* < 0.001 indicate statistically significant differences relative to the vehicle-treated control group.

The methylation of each histone H3 lysine residue is catalyzed by a specific individual enzyme (e.g., SET1A/B for H3K4, EZH1/2 for H3K27, NSD1/2/3 for H3K36, or DOT1L for H3K79) [20,21]. To further confirm whether the inhibitory activity of 9l towards histone H3 lysine methylation was associated with DOT1L-catalyzed H3K79 methylation, we examined other histone H3 lysine methylated residues by western blotting. We observed that cells treated with 9l for 48 h effectively suppressed H3K79 methylations in a concentration-dependent manner, but other histone H3 methylated lysine residues were not affected by 9l treatment, indicating a selective suppression of DOT1L enzyme activity by 9l (Figure 5A). Moreover, 9l effectively inhibited DOT1L enzyme activity in a concentration-dependent manner, with a half-maximal inhibitory concentration (IC_50_) value of 362.8 nM (Figure 5B).

### 2.4. 9l Inhibits Cell Invasion and Migration by Regulating Epithelial-Mesenchymal Transition (EMT) Biomarker Expression in Human TNBC Cells

**Figure 6:**
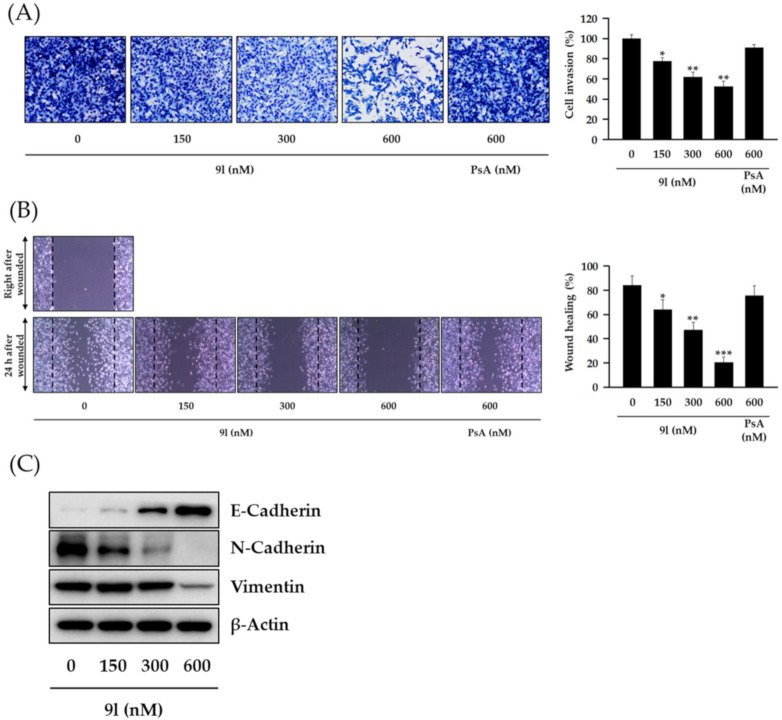
The inhibitory effects of 9l on cell invasion and migration via EMT gene regulation in TNBC cells. (**A**) MDA-MB-231 cells were pretreated with 9l or PsA at the indicated concentrations for 24 h, reseeded into the upper chambers of Transwell inserts, and incubated for 24 h. Cells invading the lower chambers were fixed, stained, imaged (**left**), and counted (**right**). * *p* < 0.05 and ** *p* < 0.01 values indicated statistically significant differences relative to the vehicle-treated control group. (**B**) Monolayers of MDA-MB-231 cells were mechanically scratched and treated with 9l or PsA for 24 h. Representative light microscopy images of wound closure are shown (**left**). Wound areas were quantified using Image J (**right**). * *p* < 0.05, ** *p* < 0.01 and *** *p* < 0.001 values indicated statistically significant differences relative to the vehicle-treated control group. (**C**) The protein expression levels of E-cadherin, N-cadherin, and vimentin in MDA-MB-231 cells treated with 9l for 24 h were determined via western blotting. β-Actin was used as an internal control.

Approximately 30% of BCs metastasize from the primary cancer site to distant sites, e.g., bone, lung, liver, and brain via the lymphatic system and bloodstream, translating to a survival rate of approximately 20% for metastatic BC [22]. Due to these aggressive characteristics, TNBCs are highly metastatic when compared with typical BC, and thus metastasis is the major cause of death in TNBC patients. Recent reports have indicated that aberrantly regulated H3K79 methylation in TNBC is correlated with increased metastatic potential [23]. Since cancer metastasis occurs concomitantly with a series of biological processes, including cell invasion and migration, we speculated whether 9l could suppress the metastatic potential of TNBC cells by inhibiting these key processes. As shown in Figure 6A,B, 9l significantly inhibited cell invasion and wound closure (cell migration) in a concentration-dependent manner in MDA-MB-231 cells. Further experiments revealed that the regulation of invasion and migration by **9l** was correlated with the modulation of EMT biomarker including, E-cadherin upregulation, and N-cadherin and vimentin downregulation (Figure 6C).

### 2.5. Antitumor Activity of 9l in MDA-MB-231/Luc Cells-Implanted Orthotopic Mouse Model

**Figure 7:**
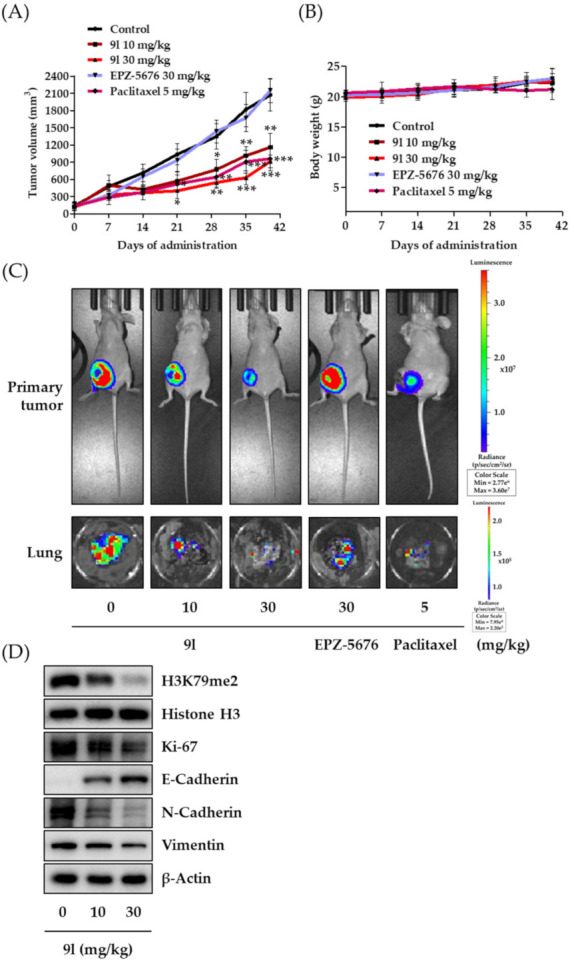
Inhibitory activity of 9l on tumor growth and pulmonary metastasis in an orthotopic mouse model, implanted with MDA-MB-231/Luc cells. (**A**) MDA-MB-231/Luc cells (1 × 10^7^ cells/mouse) were injected orthotopically into the fourth fat pad of female BALB/c nude mice. 9l (10 or 30 mg/kg body weight), EPZ-5676 (30 mg/kg body weight), or paclitaxel (5 mg/kg body weight) were administered intraperitoneally, three times a week for 38 days. Tumor volumes were measured every seven days using an electronic caliper. * *p* < 0.05, ** *p* < 0.01 and *** *p* < 0.001 were compared with the vehicle-administered control group. (**B**) Mice body weights were measured to assess general toxicity. (**C**) Luciferase activity imaging of BALB/c nude mice on the last day of the experiment. The lungs of the mice from each study group were excised at the termination of the experiment and imaged using IVIS. (**D**) Small portions of tumor from each group were homogenized in complete lysis buffer (Active Motif), and the protein expression levels of H3K79me2, Ki-67, E-cadherin, N-cadherin, and vimentin were determined via western blotting. Histone H3 and β-actin were used as internal controls.

The antitumor activity of 9l was evaluated using an orthotopic mouse metastasis model implanted with luciferase-expressing MDA-MB-231 cells. The cells (1 × 10^7^ cells/mouse) were surgically inoculated into fourth fat pad of female nude mice. After tumor size reached approximately 130 mm^3^, 9l (10 or 30 mg/kg body weight), EPZ-5676 (30 mg/kg body weight), or paclitaxel (5 mg/kg body weight) was intraperitoneally administered to each group, three times per week for 38 days. In our previous study for antitumor activity of PsA analogs against lung cancer and TNBC cells, we found that the administration dose of 30 mg/kg of the PsA analogs was effective without overall toxicity [17,18]. Therefore, in the present study, we also primarily selected the doses of 10 and 30 mg/kg/body weight to determine the effect of compound 9l on the tumor growth in an in vivo orthotopic mouse model. When compared with the vehicle-administered control group, 9l significantly inhibited tumor growth. At the end of the experiment period, inhibition rates were 44.0% and 55.8% at 10 mg/kg and 30 mg/kg, respectively (Figure 7A). Consistent with a previous report, the pre-developed DOT1L inhibitor, EPZ-5676, did not affect tumor growth in mice. No overt toxicity or changes in body weight were found in groups treated with 9l when compared with the control group, while moderate body weight loss occurred in the group of paclitaxel treatment (Figure 7B). Optical images taken by IVIS also identified tumor growth and lung metastasis incidence in mice. As compared to vehicle-treated control group, the luminescence signal intensity from the primary tumor sites was decreased in 9l-treatment groups, and the luminescence signal intensity in excised lung tissues was also efficiently suppressed by 9l treatment (Figure 7C). Additionally, biochemical analyses of primary tumor tissues identified protein expression correlations between the antitumor activity of 9l and its EMT regulatory effects via DOT1L-catalyzed H3K79 methylation. We observed that H3K79me2, N-cadherin, and vimentin protein levels were downregulated, while E-cadherin levels were upregulated in excised tumors in the 9l-administered group. Moreover, 9l significantly suppressed Ki-67 expression, a representative proliferation marker in tumor tissues (Figure 7D). These data confirmed that 9l effectively suppressed tumor growth and lung metastasis of TNBC cells. Thus, the antitumor activity of 9l may be associated with the selective regulation of histone methylation and EMT processes.

## 3. Discussion

Hormonal therapies are the most effective strategies for treating hormone receptor-positive BCs. Despite their efficacy, these drugs are limited due to the absence of each receptor [24,25]. Of the BCs, TNBC is considered to be the most aggressive. Due to limited treatment options, conventional chemotherapeutic agents such as taxanes or anthracyclines are often used to treat TNBC patients in the clinic; however, these chemotherapeutic agents exhibit relatively low selectivity, thus causing severe side-effects [26,27].

The oncogenic role of HMTs is associated with the aberrant expression and activation of its associated signaling pathways in several cancers [28]. Each HMT catalyzes a specific amino acid residue of histone residues, and further regulates downstream target genes involved in cancer initiation, proliferation, metastasis, and angiogenesis [29,30,31]. DOT1L, a methyltransferase that catalyzes the methylation of histone H3 lysine 79 residues, is known to promote tumorigenesis in *MLL*-rearranged leukemia, with aberrant expression related to poor clinical outcomes. Thus, several nucleoside-class DOT1L inhibitors have been developed, and have culminated in a clinical trial investigating leukemia harboring rearrangements in the *MLL* gene [32]. However, precise mechanisms of how DOT1L stimulates solid tumor progression and metastasis requires comprehensive investigation, and similarly more effective DOT1L inhibitors for solid tumor treatment are also required.

In our previous study, we proposed a novel, precise synthesis method for PsA and its analogs and investigated that a β-naphthyl substituted homodimeric analog PsA-111 exhibits HDAC inhibitory activity in lung cancer cells [17]. To further develop novel epigenetic regulators for intractable cancers, we revealed that PsA-3091, a tertiary butyl substituted heteromonomeric disulfide structured analog, showed DOT1L-dependent antitumor activity towards TNBC cells [18]. Based on these findings, we concluded that homodimeric structured analogs were more effective for HDAC inhibition, just like PsA, while novel heteromonomeric structured analogs were effective towards DOT1L inhibition. In this study, as a part of our ongoing efforts to develop DOT1L inhibitors against TNBCs, we designed and synthesized novel selenium-bearing heteromonomeric structured PsA analogs. To design the chemically stable selenium-bearing analogs, we primarily confirm the chemical stability of the analogs by thin layer chromatography (TLC) analysis of compound 9 (Figure 2, Se-S analog). As shown in the TLC pattern, we could not detect decomposed spots by using compound 9, indicating that the Se-S bond analog is chemically stable (Figure S1). Based on the findings, further Se-S analogs (half-selenopsammaplin A, HSPA analogs) were designed and synthesized. We observed that the tertiary butyl substituted HSPA analog, 9l, exhibited the most selective growth inhibition against TNBC cells, along with effective inhibition of DOT1L activity. When compared with our previously reported PsA-3091 analog, 9l exhibited improved inhibitory activity not only against TNBC cell growth, but also against DOT1L activity. As a potential DOT1L inhibitor, 9l effectively suppressed the methylation of histone H3 lysine 79 residues. Similarly, 9l also effectively inhibited the invasive and migration potential of MDA-MB-231 cells along with the regulation of EMT processes, which is associated with cancer metastasis. In addition, 9l exhibited significant antitumor activity in an MDA-MB-231/Luc-implanted orthotopic mouse model. While the precise mechanism of how DOT1L activity is regulated by 9l and whether any metabolite affects the biological activity will be explored in future studies, 9l appears to be a promising lead compound in the development of novel DOT1L inhibitors for metastatic TNBC treatment.

In summary, we demonstrated that the histone methyltransferase DOT1L plays a key role in the regulation of tumor growth and metastasis in TNBC cells. Furthermore, 9l, a synthetic heteromonomeric Se-S analog of PsA, was characterized as a novel DOT1L inhibitor with considerable antitumor activity. We hypothesize the underlying mechanism of action for 9l in MBA-MB-231 TNBC cells involves the inhibition of DOT1L-mediated H3K79 methylation, and EMT pathway regulation. Therefore, these findings are clinically significant for two reasons; ours is the first report identifying synthetic methods for generating novel HSPA analogs, and targeting DOT1L via 9l may be a compelling strategy for treating aggressive and metastatic TNBC.

## 4. Materials and Methods

### 4.1. Chemistry

All reagents purchased from commercial sources were used without further purification. Infrared analyses (KBr pellet) were performed by FT-IR. ^1^H-NMR spectra were recorded at 400 MHz or 800 MHz with reference to CH_3_OH (δ 3.31). ^13^C-NMR spectra were obtained by 100 MHz spectrometer relative to the central CD_3_OD (δ 49.0) resonance. Coupling constants (*J*) in ^1^H-NMR are in Hz. Low-resolution mass spectra (LRMS) and high-resolution mass spectra (HRMS) were measured on positive-ion FAB, CI, or Q-TOF (ESI) spectrometers.

**General Procedure for the Synthesis of selenopsammaplin A monomer Analogs (23~36).**

***(E)-3-(3-Bromo-4-hydroxyphenyl)-2-(hydroxyimino)-N-(2-((phenylthio)selanyl)ethyl)propanamide (*9a*).*** To a solution of selenopsammaplin A (**5a**, 443 mg, 0.58 mmol) and diphenyldisulfide (128 mg, 0.58 mmol) in DMSO (12 mL) and 0.5 M phosphate buffer (pH 8.3, 4 mL) was added dithiothreitol (DTT, 9 mg, 0.058 mmol). The reaction mixture was stirred at room temperature for 5 h. The reaction mixture was diluted with ethyl acetate (50 mL), washed with H_2_O (5 mL × 5) and brine (5 mL), dried over anhydrous MgSO_4_, concentrated in vacuo. The residue was purified by column chromatography (silica gel, dichloromethane:methanol = 20:1) to afford **9a** (160 mg, 56% yield) as a yellow caramel. ^1^H-NMR (400 MHz, CD_3_OD): δ 7.50–7.53 (m, 2H), 7.34 (d, *J* = 1.8 Hz, 1H), 7.24 (dd, *J*_1_ = 8.3 Hz, *J*_2_ = 6.9 Hz, 2H), 7.18 (d, *J* = 7.4 Hz, 1H), 7.04 (dd, *J*_1_ = 8.3 Hz, *J*_2_ = 2.3 Hz, 1H), 6.73 (d, *J* = 8.3 Hz, 1H), 3.75 (s, 2H), 3.57 (t, *J* = 6.9 Hz, 2H), 3.01 (t, *J* = 6.9 Hz, 2H) ppm; ^13^C-NMR (100 MHz, CD_3_OD) δ 165.94, 153.96, 153.17, 138.15, 134.67, 131.08, 130.68, 130.56, 130.28, 128.43, 117.11, 110.62, 40.19, 31.72, 28.79 ppm; FT/IR = 3380, 3058, 2925, 2864, 1658, 1626, 1578, 1531, 1493, 1438, 1358, 1283, 1210, 1044, 1010, 968, 822, 801, 742, 689, 615 cm^−1^; HRMS (FAB) [M + H]^+^ calcd. for [C_17_H_18_BrN_2_O_3_SSe]^+^ 488.9387, found: 488.9384.

***(E)-2-(Hydroxyimino)-3-phenyl-N-(2-((phenylthio)selanyl)ethyl)propanamide (*9b*).*** Following the synthetic procedure of **9a**, compound **9b** was obtained from compound **5b** (100 mg, 0.176 mmol) as a pale colorless caramel (35 mg, 48% yield). ^1^H-NMR (400 MHz, CD_3_OD): δ 7.50–7.53 (m, 2H), 7.23 (d, *J* = 2.3 Hz, 1H), 7.21 (dd, *J*_1_ = 8.0 Hz, *J*_2_ = 6.6 Hz, 2H), 7.17 (t, *J* = 7.3 Hz, 1H), 7.01 (dd, *J*_1_ = 8.5 Hz, *J*_2_ = 2.1 Hz, 1H), 6.71 (d, *J* = 8.2 Hz, 1H), 3.74 (s, 2H), 3.55 (t, *J* = 7.1 Hz, 2H), 2.99 (t, *J* = 6.9 Hz, 2H) ppm; ^13^C-NMR (100 MHz, CD_3_OD) δ 166.05, 153.27, 138.26, 138.15, 131.08, 130.27, 130.23, 129.44, 128.43, 127.36, 40.22, 31.71, 30.06ppm; FT/IR = 3277, 3060, 2925, 2855, 1659, 1627, 1579, 1528, 1495, 1475, 1438, 1359, 1299, 1208, 1140, 1011, 968, 788, 741, 700 cm^−1^; HRMS (FAB) [M + H]^+^ calcd. for [C_17_H_19_N_2_O_2_SSe]^+^ 395.0332, found: 395.0333.

***(E)-2-(Hydroxyimino)-3-(naphthalen-2-yl)-N-(2-((phenylthio)selanyl)ethyl)propanamide (*9c*).*** Following the synthetic procedure of **9a**, compound **9c** was obtained from compound **5c** (28.2 mg 0.043 mmol) as a pale colorless caramel (10.1 mg, 52% yield). ^1^H-NMR (400 MHz, CD_3_OD): δ 7.50–7.53 (m, 2H), 7.23 (d, *J* = 2.3 Hz, 1H), 7.21 (dd, *J*_1_ = 8.0 Hz, *J*_2_ = 6.6 Hz, 2H), 7.02 (t, *J* = 7.3 Hz, 1H), 6.91 (dd, *J*_1_ = 8.5 Hz, *J*_2_ = 2.1 Hz, 1H), 6.70 (d, *J* = 8.2 Hz, 1H), 3.74 (s, 2H), 3.55 (t, *J* = 7.1 Hz, 2H), 2.98 (t, *J* = 6.9 Hz, 2H) ppm; ^13^C-NMR (100 MHz, CD_3_OD) δ 166.1, 153.2, 138.1, 135.8, 135.2, 133.8, 131.1, 130.4, 130.2, 129.0, 128.8, 128.7, 128.5, 128.4, 127.1, 126.5, 40.2, 31.8, 30.3 ppm; FT/IR = 3283, 3055, 2925, 2853, 1657, 1626, 1578, 1529, 1475, 1438, 1360, 1271, 1213, 1011, 971, 861, 817, 792, 742, 688 cm^−1^; HRMS (FAB) [M + H]^+^ calcd. for [C_21_H_21_N_2_O_2_SSe]^+^ 445.0489, found: 445.0489.

***(E)-3-(4-Fluorophenyl)-2-(hydroxyimino)-N-(2-((phenylthio)selanyl)ethyl)propanamide (*9d*).*** Following the synthetic procedure of **9a**, compound **9d** was obtained from compound **5d** (47.1 mg 0.078 mmol) as a pale yellow caramel (14.9 mg, 47% yield). ^1^H-NMR (400 MHz, CD_3_OD): δ 7.50–7.53 (m, 2H), 7.09–7.27 (m, 8H), 3.87 (d, *J* = 5.1 Hz, 2H), 3.56 (t, *J* = 6.9 Hz, 2H), 2.99 (t, *J* = 6.9 Hz, 2H) ppm; ^13^C-NMR (100 MHz, CD_3_OD) δ 165.92, 164.3, 161.8, 153.1, 138.2, 134.2, 134.2, 132.0, 131.9, 131.1, 130.3, 128.4, 116.1, 115.9, 40.2, 31.73, 29.3 ppm; FT/IR = 3275, 3058, 2926, 1658, 1626, 1578, 1529, 1508, 1476, 1438, 1158, 1092, 1014, 969, 821, 795, 741, 688 cm^−1^; HRMS (FAB) [M + H]^+^ calcd. for [C_17_H_18_FN_2_O_2_SSe]^+^ 413.0238, found: 413.0238.

***(E)-3-(3,4-Difluorophenyl)-2-(hydroxyimino)-N-(2-((phenylthio)selanyl)ethyl)propanamide (*9e*).*** Following the synthetic procedure of **9a**, compound **9e** was obtained from compound **5e** (30 mg 0.046 mmol) as a pale yellow solid (10 mg, 50% yield). ^1^H-NMR (400 MHz, CD_3_OD): δ 7.51–7.53 (m, 2H), 7.16–7.27 (m, 5H), 6.91 (t, *J* = 9.0 Hz, 2H), 3.84 (s, 2H), 3.57 (t, *J* = 6.9 Hz, 2H), 3.01 (t, *J* = 7.1 Hz, 2H) ppm; ^13^C-NMR (100 MHz, CD_3_OD) δ 165.7, 152.5, 138.2, 135.8, 131.1, 130.3, 128.4, 126.8, 126.7, 126.7, 119.2, 119.0, 118.1, 118.0, 40.2, 31.7, 29.3 ppm; FT/IR = 3279, 3058, 2925, 2855, 1659, 1578, 1518, 1436, 1360, 1282, 1210, 1137, 1116, 1012, 971, 796, 771, 741, 688 cm^−1^; HRMS (FAB) [M + H]^+^ calcd. for [C_17_H_17_F_2_N_2_O_2_SSe]^+^ 431.0144, found: 431.0144.

***(E)-3-(4-Chlorophenyl)-2-(hydroxyimino)-N-(2-((phenylthio)selanyl)ethyl)propanamide (*9f*).*** Following the synthetic procedure of **9a**, compound **9f** was obtained from compound **5f** (30 mg 0.047 mmol) as a pale yellow caramel (13 mg, 65% yield). ^1^H-NMR (400 MHz, CD_3_OD): δ 7.51–7.53 (m, 2H), 7.17–7.27 (m, 7H), 3.84 (s, 2H), 3.57 (t, *J* = 6.9 Hz, 2H), 3.01 (t, *J* = 6.9 Hz, 2H) ppm; ^13^C-NMR (100 MHz, CD_3_OD) δ 165.8, 152.8, 138.2, 137.1, 133.2, 131.9, 131.1, 130.3, 129.5, 128.4, 40.2, 31.7, 30.9, 30.6, 29.5, 14.6 ppm; FT/IR = 3284, 3058, 2925, 2856, 1660, 1627, 1578, 1529, 1491, 1476, 1438, 1360, 1208, 1092, 1015, 969, 804, 741, 688 cm^−1^; HRMS (FAB) [M + H]^+^ calcd. for [C_17_H_18_ClN_2_O_2_SSe]^+^ 428.9943, found: 428.9940.

***(E)-3-(3,4-Dichlorophenyl)-2-(hydroxyimino)-N-(2-((phenylthio)selanyl)ethyl)propanamide (*9g*).*** Following the synthetic procedure of **9a**, compound **9g** was obtained from compound **5g** (46.2 mg 0.065 mmol) as a pale yellow caramel (15.4 mg, 51% yield). ^1^H-NMR (400 MHz, CD_3_OD): δ 7.52 (d, *J* = 7.4 Hz, 2H), 7.15–7.35 (m, 7H), 3.83 (s, 2H), 3.57 (t, *J* = 6.9 Hz, 2H), 3.01 (t, *J* = 6.9 Hz, 2H) ppm; ^13^C-NMR (100 MHz, CD_3_OD) δ 165.6, 152.2, 139.2, 138.2, 133.1, 132.3, 131.5, 131.2, 131.1, 130.3, 130.3, 128.4, 40.2, 31.8, 29.3 ppm; FT/IR = 3281, 3058, 2925, 2854, 1659, 1626, 1578, 1529, 1470, 1438, 1398, 1359, 1302, 1206, 1132, 1022, 970, 874, 817, 781, 741, 687 cm^−1^; HRMS (FAB) [M + H]^+^ calcd. for [C_17_H_17_Cl_2_N_2_O_2_SSe]^+^ 462.9553, found: 462.9548.

***(E)-3-(4-Bromophenyl)-2-(hydroxyimino)-N-(2-((phenylthio)selanyl)ethyl)propanamide (*9h*).*** Following the synthetic procedure of **9a**, compound **9h** was obtained from compound **5h** (30 mg 0.041 mmol) as a pale yellow caramel (9.6 mg, 48% yield). ^1^H-NMR (400 MHz, CD_3_OD): δ 7.51–7.53 (m, 2H), 7.04–7.27 (m, 6H), 3.84 (s, 2H), 3.58 (t, *J* = 6.9 Hz, 2H), 3.02 (t, *J* = 7.1 Hz, 2H) ppm; ^13^C-NMR (100 MHz, CD_3_OD) δ 165.8, 152.7, 138.2, 137.6, 132.5, 132.3, 131.1, 130.3, 128.4, 121.1, 40.2, 31.7, 29.6, 14.6 ppm; FT/IR = 3226, 3058, 2925, 2854, 1658, 1627, 1578, 1529, 1487, 1438, 1359, 1297, 1207, 1097, 1070, 1011, 969, 800, 741, 691, 666 cm^−1^; HRMS (FAB) [M + H]^+^ calcd. for [C_17_H_18_BrN_2_O_2_SSe]^+^ 472.9438, found: 472.9435.

***(E)-3-(4-Ethoxyphenyl)-2-(hydroxyimino)-N-(2-((phenylthio)selanyl)ethyl)propanamide (*9i*).*** Following the synthetic procedure of **9a**, compound **9i** was obtained from compound **5i** (133 mg 0.2 mmol) as a pale colorless caramel (35.4 mg, 40% yield). ^1^H-NMR (400 MHz, CD_3_OD): δ 7.50–7.53 (m, 2H), 7.40 (d, *J* = 2.3 Hz, 1H), 7.34 (d, *J* = 8.2 Hz, 1H), 7.23–7.27 (m, 2H), 7.16–7.20 (m, 2H), 3.84 (s, 2H), 3.58 (t, *J* = 6.9 Hz, 2H), 3.02 (t, *J* = 6.9 Hz, 2H) ppm; ^13^C-NMR (100 MHz, CD_3_OD) δ 166.2, 159.0, 153.7, 138.2, 131.3, 131.1, 130.4, 130.3, 130.1, 128.9, 128.6, 128.4, 115.5, 64.6, 41.2, 40.2, 31.7, 29.5, 29.2, 15.3 ppm; FT/IR = 3285, 3058, 2976, 2926, 1658, 1626, 1579, 1509, 1476, 1438, 1359, 1300, 1245, 1178, 1116, 1047, 1011, 969, 922, 792, 741, 689 cm^−1^; HRMS (FAB) [M + H]^+^ calcd. for [C_19_H_23_N_2_O_3_SSe]^+^ 439.0595, found: 439.0595.

***(E)-3-(4-(Benzyloxy)phenyl)-2-(hydroxyimino)-N-(2-((phenylthio)selanyl)ethyl)propanamide (*9j*).*** Following the synthetic procedure of **9a**, compound **9j** was obtained from compound **5j** (80 mg 0.1 mmol) as a pale colorless caramel (22.8 mg, 44% yield). ^1^H-NMR (400 MHz, CD_3_OD): δ 7.51–7.53 (m, 2H), 7.12–7.27 (m, 5H), 6.73 (d, *J* = 8.7 Hz, 2H), 3.94 (q, *J* = 7.0 Hz, 2H), 3.79 (s, 2H), 3.56 (t, *J* = 7.1 Hz, 2H), 2.99 (t, *J* = 7.1 Hz, 2H), 1.32 (t, *J* = 6.9 Hz, 3H) ppm; ^13^C-NMR (100 MHz, CD_3_OD) δ 166.14, 166.06, 158.89, 153.69, 153.58, 139.02, 138.25, 138.18, 131.32, 131.09, 130.51, 130.39, 130.28, 129.61, 128.94, 128.86, 128.68, 128.63, 128.43, 115.91, 71.10, 41.22, 40.22, 31.74, 30.94, 30.65, 30.35, 29.48, 29.18, 23.92, 14.63 ppm; FT/IR = 3378, 3219, 3063, 2923, 1650, 1624, 1531, 1509, 1454, 1438, 1382, 1291, 1252, 1178, 1106, 1045, 1011, 797, 730, 692 cm^−1^; HRMS (FAB) [M + H]^+^ calcd. for [C_24_H_25_N_2_O_3_SSe]^+^ 501.0751, found: 501.0752.

***(E)-2-(Hydroxyimino)-3-(4-nitrophenyl)-N-(2-((phenylthio)selanyl)ethyl)propanamide (*9k*).*** Following the synthetic procedure of **9a,** compound **9k** was obtained from compound **5k** (30 mg 0.046 mmol) as a pale yellow solid (9.2 mg, 48% yield). ^1^H-NMR (400 MHz, CD_3_OD): δ 7.52 (dt, *J*_1_ = 7.0 Hz, *J*_2_ = 1.4 Hz, 2H), 7.14–7.39 (m, 9H), 6.82 (dd, *J*_1_ = 6.7 Hz, *J*_2_ = 2.1 Hz, 2H), 4.98 (d, *J* = 12.4 Hz, 2H), 3.80 (d, *J* = 4.1 Hz, 2H), 3.55 (t, *J* = 6.9 Hz, 2H), 2.99 (t, *J* = 6.9 Hz, 2H) ppm; ^13^C-NMR (100 MHz, CD_3_OD) δ 165.5, 152.0, 148.1, 146.4, 138.2, 131.3, 131.1, 130.4, 130.3, 128.9, 128.6, 128.5, 124.6, 40.3, 31.8, 30.2 ppm; FT/IR = 3222, 3058, 2925, 1659, 1627, 1604, 1578, 1519, 1475, 1438, 1345, 1209, 1143, 1109, 1014, 970, 860, 816, 741, 712, 689 cm^−1^; HRMS (FAB) [M + H]^+^ calcd. for [C_17_H_18_N_3_O_4_SSe]^+^ 440.0183, found: 440.0183.

***(E)-3-(4-(tert-Butyl)phenyl)-2-(hydroxyimino)-N-(2-((phenylthio)selanyl)ethyl)propanamide (*9l*).*** Following the synthetic procedure of **9a**, compound **9l** was obtained from compound **5l** (26.2 mg 0.038 mmol) as a pale colorless caramel (8.1 mg, 46% yield). ^1^H-NMR (400 MHz, CD_3_OD): δ 8.06–8.09 (m, 2H), 7.46–7.53 (m, 4H), 7.17–7.27 (m, 3H), 4.00 (d, *J* = 2.8 Hz, 2H), 3.58 (t, *J* = 6.9 Hz, 2H), 3.02 (t, *J* = 6.9 Hz, 2H) ppm; ^13^C-NMR (100 MHz, CD_3_OD) δ 166.15, 153.48, 150.26, 138.17, 135.10, 131.08, 130.27, 129.92, 128.42, 126.33, 40.22, 35.32, 31.97, 31.74 ppm; FT/IR = 3282, 3057, 2961, 2867, 2520, 1657, 1628, 1578, 1527, 1475, 1437, 1362, 1269, 1210, 1143, 1110, 1022, 969, 836, 812, 741, 688 cm^−1^; FT/IR = 3282, 3057, 2961, 2867, 2520, 1657, 1628, 1578, 1527, 1475, 1437, 1362, 1269, 1210, 1143, 1110, 1022, 969, 836, 812, 741, 688 cm^−1^; HRMS (FAB) [M + H]^+^ calcd. for [C_21_H_27_N_2_O_2_SSe]^+^ 451.0958, found: 451.0959.

***(E)-3-(3-Chloro-4-hydroxyphenyl)-2-(hydroxyimino)-N-(2-((phenylthio)selanyl)ethyl)propanamide (*9m*).*** Following the synthetic procedure of **9a**, compound **9m** was obtained from compound **5m** (80 mg 0.1 mmol) as a pale yellow caramel (19.8 mg, 38% yield). ^1^H-NMR (400 MHz, CD_3_OD): δ 7.51–7.53 (m, 2H), 7.34 (d, *J* = 2.3 Hz, 1H), 7.25 (t, *J* = 7.5 Hz, 2H), 7.17 (t, *J* = 7.3 Hz, 1H), 7.04 (dd, *J* = 8.5, 2.1 Hz, 1H), 6.73 (d, *J* = 8.2 Hz, 1H), 3.75 (s, 2H), 3.57 (t, *J* = 7.1 Hz, 2H), 3.01 (t, *J* = 6.9 Hz, 2H) ppm; ^13^C-NMR (100 MHz, CD_3_OD) δ 165.95, 153.91, 153.19, 138.17, 134.67, 131.10, 130.73, 130.57, 130.27, 128.43, 117.11, 110.62, 40.20, 31.75, 28.80 ppm; FT/IR = 3328, 3059, 2925, 2853, 1732, 1659, 1627, 1578, 1529, 1511, 1498, 1458, 1438, 1375, 1274, 1213, 1056, 1022, 969, 887, 823, 801, 742, 688, 607 cm^−1^; HRMS (FAB) [M + H]^+^ calcd. for [C_17_H_18_ClN_2_O_3_SSe]^+^ 444.9892, found: 444.9890.

***(E)-3-(3-Fluoro-4-hydroxyphenyl)-2-(hydroxyimino)-N-(2-((phenylthio)selanyl)ethyl)propanamide (*9n*).*** Following the synthetic procedure of **9a**, compound **9n** was obtained from compound **5n** (30 mg 0.04 mmol) as a pale yellow caramel (8.2 mg, 40% yield). ^1^H-NMR (400 MHz, CD_3_OD): δ 7.67–7.76 (m, 4H), 7.48–7.50 (m, 2H), 7.36–7.41 (m, 3H), 7.19–7.23 (m, 2H), 7.13–7.15 (m, 1H), 4.04 (s, 2H), 3.57 (t, *J* = 6.9 Hz, 2H), 3.00 (t, *J* = 6.9 Hz, 2H), 1.26 (s, 1H) ppm; ^13^C-NMR (100 MHz, CD_3_OD) δ 165.81, 153.82, 153.04, 138.02, 134.53, 130.95, 130.55, 130.42, 130.14, 128.29, 116.98, 110.49, 49.66, 49.44, 49.23, 49.02, 48.81, 48.59, 48.37, 40.06, 31.59, 28.65 ppm; FT/IR = 3059, 2925, 2855, 2320, 1877, 1601, 1578, 1491, 1476, 1360, 1143, 1111, 1901, 1066, 970, 918, 878, 802, 720, 664, 633 cm^−1^; HRMS (FAB) [M + H]^+^ calcd. for [C_17_H_18_FN_2_O_3_SSe]^+^ 429.0187, found: 429.0188.

### 4.2. DOT1L Enzyme Activity Assay

DOT1L enzyme activity was measured using 5 μM S-adenosyl methionine (SAM) as the methyl group donor, synthesized DOT1L substrate as the substrate, and 25 ng/μL DOT1L enzymes from BPS Bioscience (Cat. No. 52202; San Diego, CA, USA) according to the manufacturer’s instructions.

### 4.3. Cell Culture and Chemicals

Human breast epithelial cell line (MCF10A) and human TNBC cell lines (MDA-MB-468, MDA-MB-231, Hs578T, and HCC38 cells) were provided by the American Type Culture Collection (Manassas, VA, USA). MCF10A cells were cultured in Dulbecco’s Modified Eagle’s Medium/Nutrient Mixture F-12 containing 5% donor horse serum, 100 ng/mL cholera toxin, 10 µg/mL human insulin, 20 ng/mL epidermal growth factor, 0.5 µg/mL hydrocortisone, and penicillin-streptomycin (sodium penicillin G: 100 units/mL; streptomycin: 100 μg/mL). The TNBC cells were cultured in medium [Dulbecco’s Modified Eagle’s Medium for MDA-MB-468, MDA-MB-231, and Hs578T cells; Roswell Park Memorial Institute 1640 medium for HCC38 cells] supplemented with penicillin-streptomycin and 10% fetal bovine serum at 37 °C in a humidified incubator with 5% carbon dioxide. MCF10A cells were cultured in DMEM/F12 containing 5% donor horse serum, 100 ng/mL cholera toxin, 10 µg/mL human insulin, 20 ng/mL epidermal growth factor, 0.5 µg/mL hydrocortisone and penicillin-streptomycin. All reagents used for cell culture, including culture medium, fetal bovine serum, trypsin-EDTA solution (1×), and penicillin-streptomycin solution (100×), were purchased from Gibco (Grand Island, NE, USA). Laemmli sample buffer (2×) and 2-mercaptoethanol were purchased from Bio-Rad Laboratories, Inc. (Hercules, CA, USA). EPZ-5676 was purchased from Cayman Chemical (Ann Arbor, MI, USA). Dimethyl sulfoxide (DMSO), bicinchoninic acid, copper (II) sulfate solution, bovine serum albumin (BSA), trichloroacetic acid, sulforhodamine B (SRB), and paclitaxel were purchased from Sigma–Aldrich (St. Louis, MO, USA) [33].

### 4.4. Western Blotting Analysis

Total cell lysates were prepared in 2× sample loading buffer [250 mM Tris-HCl (pH 6.8), 10% glycerol, 4% sodium dodecyl sulfate (SDS), 2% β-mercaptoethanol, 0.006% bromophenol blue, 5 mM sodium orthovanadate, and 50 mM sodium fluoride; Bio-Rad, Hercules, CA, USA]. The protein concentrations of samples were quantified using the bicinchoninic acid (BCA) method [34] and a BCA Protein Assay Kit (Thermo Fisher Scientific, Waltham, MA, USA). Equal amounts of protein (5–20 μg) were separated by 6–13% SDS-polyacrylamide gel electrophoresis (PAGE) and transferred to polyvinylidene fluoride membranes (Millipore, Bedford, MA, USA). The membranes were blocked with 5% bovine serum albumin (Sigma-Aldrich) and then probed with anti-DOT1L, anti-H3K79me2, anti-β-Actin, anti-histone H3, anti-H3K4me2, anti-H3K27me2, anti-H3K36me2, anti-H3K79me1, anti-H3K79me3, and anti-Vimentin antibodies purchased from Cell Signaling Technology (Beverly, MA, USA) or with anti-E-cadherin and anti-N-cadherin antibodies purchased from (BD Biosciences, San Jose, CA, USA). The blots were detected using a WEST-Queen detection system (iNtRON Biotechnology, Seongnam, Korea) [35].

### 4.5. SRB Assay (Cell Proliferation Assay)

Cell proliferation was evaluated using the SRB assay [36]. Briefly, cells were seeded in 96-well plates and incubated for 30 min (for 0-day controls) or treated with test compounds for the times indicated. After incubation, the cells were fixed, dried, and stained with 0.4% (*w*/*v*) SRB in 1% (*v*/*v*) acetic acid solution. Unbound dye was removed by washing, and stained cells were dissolved in 10 mM Tris (pH 10.0). Cell proliferation was then determined by measuring the absorbance at 515 nm [37].

### 4.6. Transwell Cell Invasion Assay

Twenty-four-well Transwell membrane inserts (diameter: 6.5 mm, pore size: 8 μm; Corning, Tewksbury, MA, USA) were each coated with 10 μL of type I collagen (0.5 mg/mL, BD Biosciences, San Diego, CA, USA) and 20 μL of a 1:20 mixture of Matrigel (BD Biosciences) in PBS. After treatment with the indicated compounds for 24 h, MDA-MB-231 human TNBC cells were harvested, resuspended in serum-free medium, and plated (2 × 10^5^ cells/chamber) in the upper chambers of the Matrigel-coated Transwell inserts. Medium containing 30% FBS was used as a chemoattractant in the lower chambers. After a 24 h incubation, the cells that had migrated to the outer surfaces of the lower chambers were fixed and stained using the Diff-Quik Staining Kit (Sysmex, Kobe, Japan) and imaged using a Vectra 3.0 Automated Quantitative Pathology Imaging System (Perkin Elmer, Waltham, MA, USA). Representative images from three separate experiments are shown, and the numbers of invaded cells were counted in 5 randomly selected microscopic fields (200× magnification) [38].

### 4.7. Wound Healing Assay (Cell Migration Assay)

MDA-MB-231 human TNBC cells were grown to 90% confluence in a 6-well plate. Subsequently, each cell monolayer was artificially wounded using a Scratcher (SPL Life Sciences, Pocheon, Republic of Korea), and the detached cells were removed by washing with phosphate-buffered saline (PBS, Invitrogen Corp., Carlsbad, CA, USA). The wounded cultures were then incubated with medium containing 1% FBS and various concentrations of 9l and PsA for 24 h. The wounds were photographed at 0 and 24 h under an inverted microscope (Olympus, Tokyo, Japan). The wound areas were quantified using ImageJ software (National Institutes of Health, Bethesda, MD, USA) and presented as the percent wound healing (%) relative to the wound area at 0 h.

### 4.8. In Vivo Orthotopic Mouse Tumor Model (Mammary Fat Pad Model)

All animal experiments were conducted following the guidelines approved by the Seoul National University Institutional Animal Care and Use Committee (IACUC; permission number: SNU-180430-4-1). Female nude mice (BALB/c-nu), 4 weeks old, were purchased from Central Laboratory Animal, Inc. (Seoul, Korea) and housed under pathogen-free conditions with a 12 h light-dark schedule. Luciferase expressing MDA-MB-231 cells were inoculated orthotopically into the fourth fat pad of mice (1 × 10^7^ cells in 100 μL of 50:50 PBS/Matrigel) using a 29-gauge needle [18]. Two weeks after implantation, mice were randomized into vehicle control and treatment groups of six animals per group and were administered with vehicle (EtOH/Cremophor/Normal saline = 5:5:90), 9l (10 or 30 mg/kg body weight), EPZ-5676 (30 mg/kg body weight), or paclitaxel (5 mg/kg body weight, Sigma-Aldrich) as a positive reference control. Compounds were administered intraperitoneally three times per week for 38 days. An additional week later, anesthetized mice were positioned in IVIS (PerkinElmer, Waltham, MA, USA) and were imaged 15 min after injection of 150 mg/kg D-luciferin (Gold Biotechnology, St. Louis, MO, USA) resuspended in DPBS (*w*/*o* calcium or magnesium). Mice were euthanized and primary tumors and lungs were excised, weighed, and frozen for further biochemical analysis. The length (L), width (W), and height (H) of the tumors were also measured using a digital slide caliper (Mitutoyo, Kawasaki, Japan) once a week, and tumor volumes (mm^3^) were estimated by the formula LWH/2. Toxicity was evaluated based on body weight loss.

### 4.9. Ex Vivo Biochemical Analyses of Tumors

A portion of the frozen tumors excised from nude mice on the termination day of the experiment was homogenized using a hand-held homogenizer in complete lysis buffer (Active Motif, Carlsbad, CA, USA). Aliquots were stored at −80 °C, and the levels of protein expression in the tumor lysates were determined. The protein concentrations in the tumor lysates were measured using the Bradford assay [39].

### 4.10. Statistical Analysis

The data are presented as mean values ± standard deviations (SDs) for the indicated numbers of independently performed experiments. All data are representative of the results of at least three independent experiments. Statistical significance was analyzed using Student’s *t*-test or a one-way analysis of variance coupled with Dunnett’s *t*-test. Differences were considered statistically significant at * *p* < 0.05, ** *p* < 0.01, *** *p* < 0.001.

## Figures and Tables

**Table 1:** Chemical structures of half-selenopsammaplin A (HSPA) analogs.

HSPA Analog	Structure	HSPA Analog	Structure
**9a**	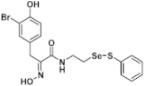	**9h**	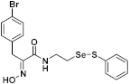
**9b**	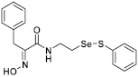	**9i**	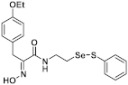
**9c**	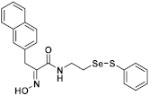	**9j**	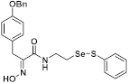
**9d**	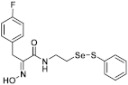	**9k**	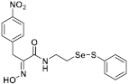
**9e**	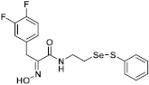	**9l**	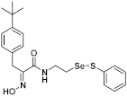
**9f**	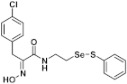	**9m**	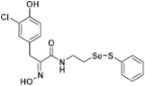
**9g**	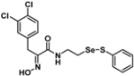	**9n**	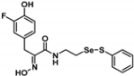

**Table 2:** Antiproliferative activities of HSPA analogs against TNBC cell line.

Analogs	MDA-MB-231 IC_50_ (μM) ^a^	MCF10A IC_50_ (μM) ^a^	Therapeutic Index ^b^
Psammaplin A (PsA)	2.82	32.70	11.60
PsA-3091	1.98	136.60	68.99
**9a**	0.21	17.24	82.10
**9b**	0.25	21.52	86.08
**9c**	0.46	28.20	61.30
**9d**	0.39	26.04	66.77
**9e**	0.43	23.32	54.23
**9f**	0.47	27.07	57.60
**9g**	0.33	22.66	68.67
**9h**	0.40	29.14	72.85
**9i**	0.17	21.02	123.65
**9j**	0.18	19.48	108.22
**9k**	0.19	18.15	95.53
**9l**	0.16	25.86	161.63
**9m**	0.25	17.02	68.08
**9n**	0.23	18.51	80.48
EPZ-5676	>50	>50	-
Paclitaxel ^c^	0.01	0.198	19.80

^a^ Results are expressed as the calculated half-maximal inhibitory concentrations (IC_50_) of test compounds (μM). ^b^ The therapeutic index was calculated as the ratio of IC50 values between MCF10A and MDA-MB-231 cells. ^c^ Paclitaxel was used as a positive control.

## Data Availability

The data presented in this study are available in this article or associated supplementary material.

## Supplementary Materials

The following are available online at https://www.mdpi.com/1424-8247/14/1/18/s1, Figure S1: Thin layer chromatography analysis.
