# Supplementary material for: Inhibition of DOT1L by Half-Selenopsammaplin A Analogs Suppresses Tumor Growth and EMT-Mediated Metastasis in Triple-Negative Breast Cancer
Source: Pharmaceuticals (Basel). 2020 Dec 28;14(1):18. doi: 10.3390/ph14010018 (PMC7824081; doi:10.3390/ph14010018)
Supplement: Supplementary file 1 [file pharmaceuticals-14-00018-s001.pdf]

Article

Inhibition of DOT1L by Half-Selenopsammaplin A Analogs Suppresses Tumor Growth and EMT-Mediated Metastasis in Triple-Negative Breast Cancer

Woong Sub Byun [1], Gyu Ho Lee [2], Hyeung-geun Park [2,*] and Sang Kook Lee [1,*]

[1] College of Pharmacy, Natural Products Research Institute, Seoul National University, Seoul 08826, Republic of Korea; sky_magic@naver.com (W.S.B.)

[2] College of Pharmacy, Research Institute of Pharmaceutical Sciences, Seoul National University, Seoul 08826, Republic of Korea; boomboombibi@snu.ac.kr (G.H.L.)

* Correspondence: hgpk@snu.ac.kr (H.-G.P.); sklee61@snu.ac.kr (S.K.L.)

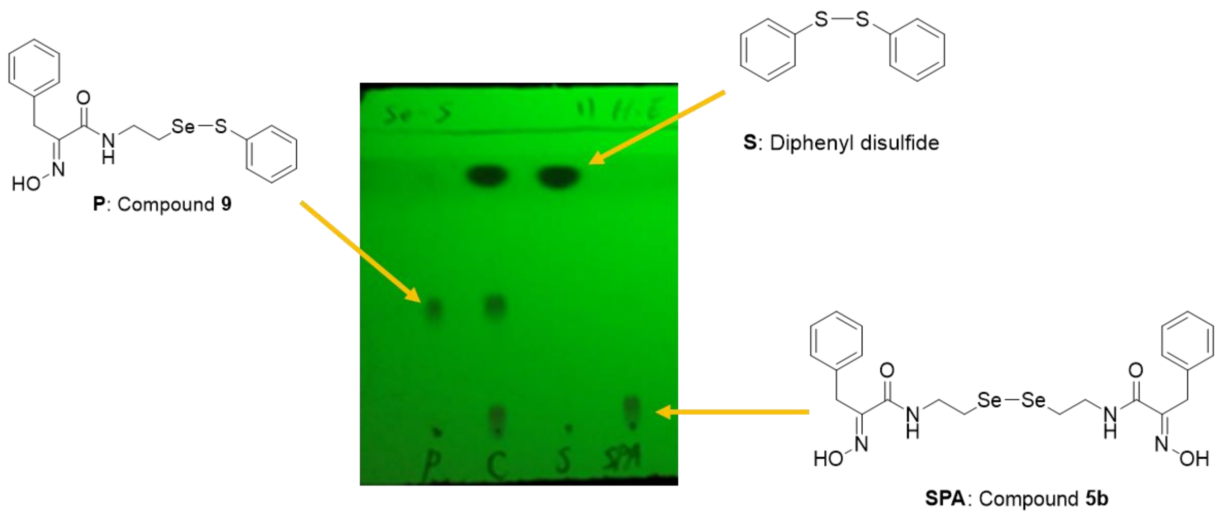

Figure S1. Thin layer chromatography analysis

Se-S bond analog (Figure 2, compound **9**) was dissolved in dichloromethane at room temperature. After 48 h, thin layer chromatography (TLC) analysis of the compound **9** was performed. (**P** : Compound **9**, **C** : Co-spot, **S** : Diphenyl disulfide, **SPA** : Compound **5b**, eluent: [Hexane:EtOAc = 1:1]).
